# Impact of Cell-of-Origin on Outcome of Patients With Diffuse Large B-Cell Lymphoma Treated With Uniform R-CHOP Protocol: A Single-Center Retrospective Analysis From North India

**DOI:** 10.3389/fonc.2021.770747

**Published:** 2021-12-02

**Authors:** Ajay Gogia, Sukesh Nair, Shalabh Arora, Lalit Kumar, Atul Sharma, Ritu Gupta, Ahitagni Biswas, Saumyaranjan Mallick

**Affiliations:** ^1^ Department of Medical Oncology, All India Institute of Medical Sciences, New Delhi, India; ^2^ Department of Laboratory Oncology, All India Institute of Medical Sciences, New Delhi, India; ^3^ Department of Radiation Oncology, All India Institute of Medical Sciences, New Delhi, India; ^4^ Department of Pathology, All India Institute of Medical Sciences, New Delhi, India

**Keywords:** survival, diffuse large B-cell lymphoma (DLBCL), rituximab, CHOP (R-CHOP), India, cell of origin

## Abstract

**Introduction:**

There is a scarcity of data from India on the impact of cell of origin (COO) on outcomes of diffuse large B-cell lymphoma (DLBCL). This study was conducted to evaluate the impact of COO on outcomes of DLBCL patients treated with uniform rituximab, cyclophosphamide, doxorubicin, vincristine, and prednisolone (RCHOP) protocol.

**Materials and Methods:**

This retrospective analysis included patients who received uniform RCHOP chemoimmunotherapy during the study period (2014–2020) at the Department of Medical Oncology at All India Institute of Medical Sciences (AIIMS), New Delhi, India. The patients were classified as germinal center B-cell like (GCB) or activated B-cell (ABC) type using the Hans classification.

**Results:**

Four hundred seventeen patients with median age of 48 years (range, 18–76) and a male-female ratio of 2:1 were included in the analysis. B symptoms and bulky disease were seen in 42.9% and 35.5%. Extranodal involvement was seen in 50.8% of cases. ECOG performance status (0-2) was present in 65%, and 51% presented with advanced disease. GCB subtype was seen in 43%, and 47% were ABC type. Low- and intermediate-risk international prognostic index (IPI) score was seen in 76% of cases. The overall response rate to RCHOP was 85.8%, including a complete response rate of 74.8%. After a median follow-up of 30 months, the 3-year event-free survival (EFS) and overall survival (OS) were 80% and 88%, respectively. The presence of B symptoms and poor ECOG performance status (3-4) was associated with inferior CR rate. Low albumin (*p* < 0.001), age >60 years (*p* = 0.001), bulky disease (*p* < 0.001), and extranodal involvement (*p* = 0.001) were associated with inferior EFS, whereas a high IPI risk score was associated with an inferior OS (*p* < 0.001). EFS and OS were not significantly different between the GCB and ABC subtypes. Grade III/IV anemia, neutropenia, and thrombocytopenia were seen in 7.6%, 13.6%, and 2.7% of patients, respectively. Febrile neutropenia was seen in 8.9% of patients, and there were four treatment-related deaths.

**Conclusions:**

Cell of origin for DLBCL has no impact on CR, EFS, and OS if patients are appropriately treated with standard doses and frequency of RCHOP. RCHOP is well tolerated in our patients, and results are comparable with the Western data.

## Introduction

Non-Hodgkin lymphoma (NHL) constitutes 2.8% of all cancer and 2.6% of all cancer-related deaths worldwide per year (1). Diffuse large B-cell lymphoma (DLBCL) is the most common NHL worldwide, constituting 25% of all cases in the Western population (2). In India, the proportion of DLBCL ranges from 34% to as high as 60% depending on the patient population selected and the classification system used (3–5). Survival of patients with DLBCL has improved significantly with the addition of rituximab (R) to the standard chemotherapy cyclophosphamide, doxorubicin, vincristine, and prednisolone (CHOP) (6). However, the use of rituximab in low- and middle-income countries (LMIC) like India is nonuniform due to resource limitations, and hence the vast majority of studies of rituximab-based treatment of DLBCL are from the Western population. Recent studies from the Indian subcontinent have shown that the proportion of rituximab-treated patients in our population is increasing (7). DLBCL can be subdivided into two prognostically important subgroups based on cell of origin (COO); germinal center B-cell like (GCB) and activated B-cell like (ABC). Prior reports have shown that survival is significantly inferior in patients with the ABC subtype (8–10). There are no studies reporting outcomes based on cell of origin in an Indian patient population managed with a uniform treatment protocol.

This study was planned to analyze the outcome of DLBCL patients treated with RCHOP and the prognostic significance of the Hans algorithm in our population. Primary objective was to assess the impact of cell-of-origin and other putative factors on event-free survival (EFS) and overall survival (OS). Secondary objectives included response to therapy and treatment toxicity.

## Materials and Methods

We accessed the medical records of patients aged 18 years or above, who underwent treatment for DLBCL between January 2014 and December 2020 at the Department of Medical Oncology, All India Institute of Medical Sciences, New Delhi, India. Patient treatment files were retrieved using International Classification of Diseases (ICD) code, and data regarding clinicopathological features and outcomes were collected. Patients who received treatment with protocols other than RCHOP (described below) were excluded from the analysis, as were patient records with significant missing data.

### Evaluation

Baseline evaluation included history and physical examination findings, complete blood counts, renal and hepatic function tests, uric acid, electrolytes, serum lactate dehydrogenase (LDH), and imaging (either contrast-enhanced computed tomography (CECT) or positron emission tomography (PET/CT)) for staging. Unilateral bone marrow aspiration and biopsy were done in all patients. Patients were also tested for human immunodeficiency virus (HIV), hepatitis B, and hepatitis C infections. Immunohistochemistry (IHC) was done on formalin-fixed paraffin-embedded sections of lymph node excision or core biopsy for confirmation of diagnosis as well as to characterize the COO using the Hans algorithm (10). Lugano staging system was used, and a size of ≥7.5 cm was used to define bulky disease. Toxicity of chemotherapy was assessed using CTCAE version 5.0. Grades 1 and 2 toxicities were often not reliably recorded in patient treatment records; hence, only grades 3 and 4 toxicities are being reported here to avoid potential under-reporting. 

### Treatment Protocol

Patients with nonbulky stages I and II disease were given either six cycles of RCHOP chemotherapy or three to four cycles of RCHOP chemotherapy followed by involved-field radiation (IFRT). Patients with bulky early-stage disease and stage III/IV disease were treated with six cycles of RCHOP chemotherapy. RCHOP consisted of rituximab at 375 mg/m^2^ on day 1, cyclophosphamide at 750 mg/m^2^ on day 1, doxorubicin at 50 mg/m^2^ on day 1, vincristine at 1.4 mg/m^2^ on day 1, and prednisolone at 100 mg on days 1–5, administered in cycles of 21 days. Radiotherapy to a dose of 36 Gy was given to patients with either bulky nodal disease or having an extranodal site of disease. Patients failing first-line therapy were offered second-line chemotherapy with rituximab, ifosfamide, carboplatin, and etoposide followed by autologous stem cell rescue.

### Outcomes

Objective responses were assessed using the Lugano response criteria for lymphoma (11). EFS was defined as the time from registration to either relapse or progression of disease or death due to any cause. Overall survival was defined as the time period from date of registration to date of death due to any cause. Patients who were lost to follow-up before any event were censored at the date of the last contact. Data were censored on December 15, 2020 for survival analysis.

### Statistical Methods

Descriptive statistics were used for describing demographic and clinical characteristics. Survival was estimated using the Kaplan-Meier method, and log-rank test was used to compare survival between groups. Cox proportional hazards model with multivariate analysis was used to identify independent predictors of outcome. Statistical analysis was done using Stata software (StataCorp. 2014. Stata Statistical Software: Release 14. College Station, TX, USA: StataCorp LP).

### Ethical Approval

This study was approved by the Institutional Ethics Committee of All India Institute of Medical Sciences, New Delhi.

## Results

During the study period, a total of 620 patients with a diagnosis of diffuse large B-cell lymphoma were registered at our center. Of these, 199 patients were excluded as they did not meet the inclusion criteria of uniform treatment with RCHOP protocol with or without radiation as described in the *Methods* section, and four patients were excluded for significant missing data. In the current analysis, we included 417 patients who received uniform RCHOP protocol with an intention-to-treat principle and had complete medical records.

### Baseline Characteristics

The median (range) age at diagnosis was 48 years (18–76), and 22% were >60 years old (**Table 1**). The male-to-female ratio was 2:1. The Eastern Cooperative Oncology Group (ECOG) performance status was 0–2 in 65% of the cases. Lymphadenopathy (LAP) was the most common presenting symptom, seen in 213 (51%), followed by B symptoms (42.9%). The most common site of LAP was cervical (47%) followed by inguinal (11%), axillary (9%), and 27% had generalized LAP (≥2 sites). Extranodal disease was present in 212 (50.8%) patients at presentation. Bone marrow and central nervous system (CNS) involvement were seen in 7.2% and 1.9% cases, respectively. Bulky disease was present in 148 patients (35.5%), while 216 (51%) patients had stage III/IV disease. International prognostic index (IPI) score was available in 397 patients (95.2%), of which 37% were low risk (IPI 0, 1), 39% were intermediate risk (2, 3), and 19% were high risk (IPI 4, 5). Using the Hans algorithm to establish the cell of origin, 43% and 47% were found to be GCB and non-GCB subtypes, while subclassification was not possible in 10% patients.

**Table 1:** Baseline characteristics of 417 diffuse large B-cell lymphoma patients.

Parameters (*N* = 417)	*n* (%)
Median age (range)	48 years (18–76)
Age >60 years	92 (22.06%)
Male:female ratio	2:1
B symptoms	179 (42.92%)
Extranodal involvement	212 (50.8%)
Ann Arbor Stage	
I	82 (20%)
II	119 (29%)
III	77 (19%)
IV	139 (32%)
Bulky disease	148 (35.5%)
ECOG performance status	
0–2	277 (65.22%)
3–4	125 (29.9%)
Missing information	20 (4.8%)
Cell of origin	
GCB	180 (43.16%)
Non-GCB	196 (47.01%)
Not classifiable	41 (9.83%)
Bone marrow involvement	30 (7.2%)
Raised LDH	191 (47%)
Hypoalbuminemia (<3.5 g/dl)	158 (38%)
International prognostic index	
Low risk	155 (37.17%)
Intermediate risk	163 (39.08%)
High risk	79 (18.94%)
Not available	20 (4.79%)
IFRT received	160 (38.36%)
Response rate	
Complete response rate	312 (74.82%)
Partial response rate	46 (11.03%)
Overall response rate	358 (85.85%)
Not evaluable/missing	17 (4.07%)

GCB, germinal center B cell; LDH, lactate dehydrogenase; IFRT, involved field radiotherapy.

### Treatment and Response

All patients received RCHOP chemotherapy with a median of six chemotherapy cycles (range, 1–8). Dose modification was required in 10% of cases because of old age and/or poor ECOG performance status, while 60 (14.38%) patients received prephase chemotherapy (consisting of cyclophosphamide at 200 mg/m^2^ once daily for 2 days plus dexamethasone at 8 mg twice daily for 5 days) before conventional RCHOP because of poor performance and high tumor bulk of disease. Grade III/IV toxicity was noted in 98 (23%) patients. The most common were neutropenia in 57 patients (13.6%), anemia in 32 patients (7.6%), and thrombocytopenia in 11 patients (2.7%). Febrile neutropenia was seen in 37 patients (8.87%). Other common grade III/IV toxicities include peripheral neuropathy, diarrhea, mucositis, vomiting, and extravasation in eight, six, five, four, and two patients, respectively. Hospital admission for toxicity was required in 38 (9%) patients mostly for febrile neutropenia and GI toxicities. Death during initial chemotherapy was seen in 10 (2.3%) patients, of which six were due to progressive disease, and the remaining due to febrile neutropenia. IFRT was used in 160 patients (38.36%), comprising 117 patients with early nonbulky disease, and 43 patients with advanced disease who received RT predominantly for bulky disease or residual disease (radiation given after nonviable tumor on repeat biopsy). The overall response rate was 85%, including complete remission (CR) seen in 312 (74.82%) patients. Thirty cases (7.2%) had primary refractory disease and six patients died of progressive disease during treatment. Presence of B symptoms [CR 65% *vs*. 83% of those with no B symptoms; odds ratio, 1.76 (1.08–2.87, *p* < 0.001)] and poor ECOG PS (67% *vs*. 81% for ECOG 0, 1; odds ratio 2.88 [1.73–4.79, *p* = 0.004)] were associated with inferior CR rate.

### Survival Outcomes and Prognostic Factors

**Figure 1:**
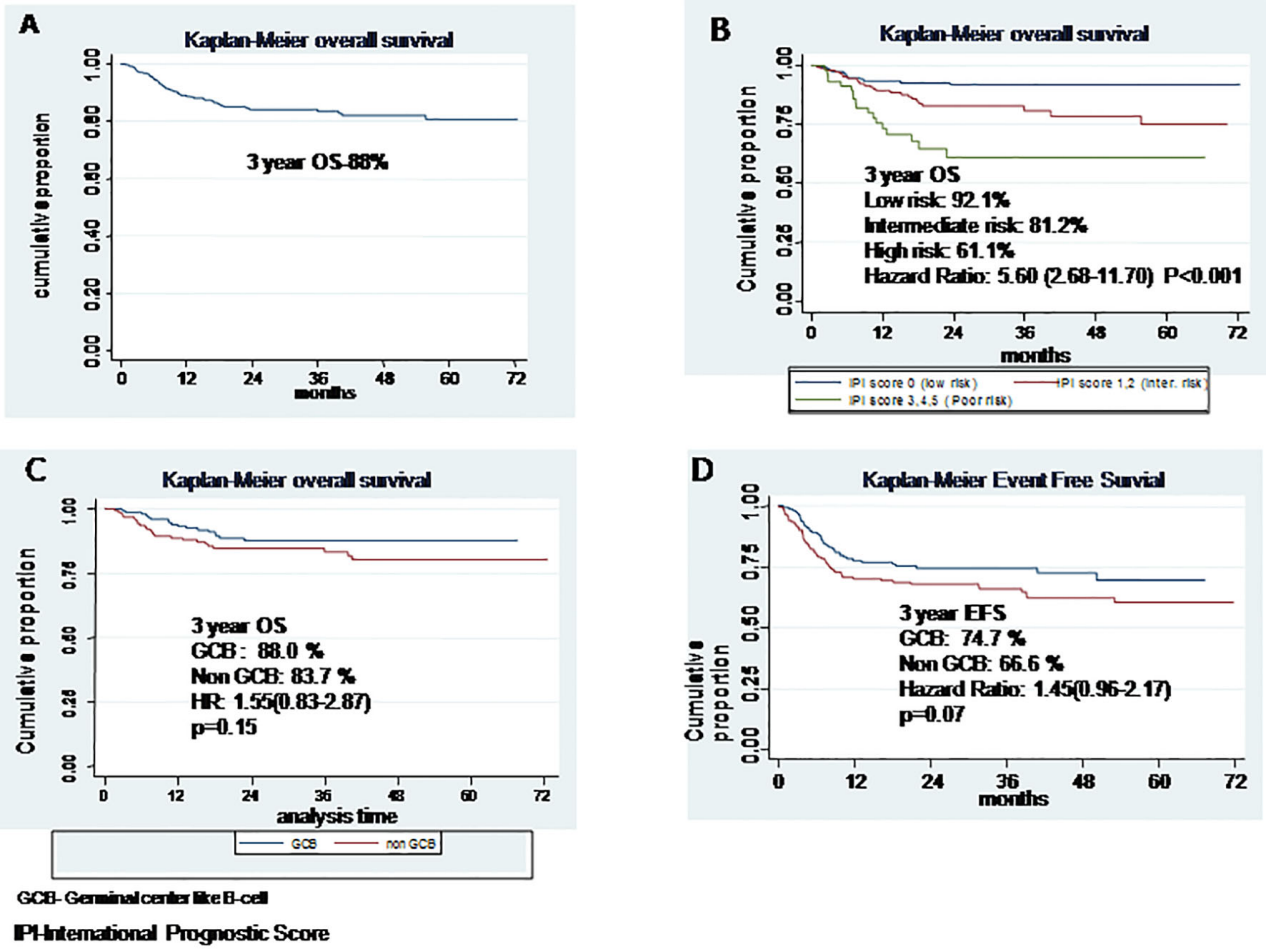
**(A)** Overall survival, **(B)** Overall survival based on International Prognostic Score, **(C)** Overall survival based on cell of origin, **(D)** Event free survival based on cell of origin.

The median follow-up time for survival analysis was 30 months (range, 6–72 months). In total, there were 63 deaths of which 25 occurred as the first event during treatment and follow-up. The median EFS and OS were not reached. Three-year cumulative EFS was 80%, and OS was 88% (**Figure 1A**) At the time of final analysis, 302 patients were alive (279 in CR) and another 42 (10.07%) were lost to follow-up of which 33 had active disease at the time of last follow-up. Of all the patients included in this analysis, 66 patients had disease relapse. Fifty-two patients opted for salvage chemotherapy, and 40 underwent autologous stem cell transplant, while those ineligible (or unwilling) for transplant were offered palliative chemotherapy (prednisolone + etoposide + procarbazine + cyclophosphamide; PEP-C) and/or best supportive care. Of the 52 patients who underwent salvage chemotherapy, 40 achieved a response (CR = 29, PR = 11) and received stem cell transplant, whereas 12 patients failed salvage therapy and resorted to palliative treatment. Nine patients had a posttransplant relapse and were offered best supportive care because of poor performance status.

**Table 2** shows the impact of various prognostic markers on survival. Age more than 60 years, presence of B symptoms, ECOG PS >2, low albumin (<3.5 g/dl), high LDH, bulky disease, and extranodal involvement were associated with inferior EFS on univariate analysis; but in multivariate analysis, age >60 years, low albumin, and bulky disease were associated with significantly inferior OS, with hazard ratios of 1.83 (1.17–2.87, *p* = 0.008), 1.63 (1.05–2.54, *p* = 0.02), and 2.94 (1.72–5.01, *p* < 0.001), respectively. Age >60 years, B symptoms, ECOG PS >2, low albumin, and bulky disease were associated with an inferior OS on univariate analysis, but in multivariate analysis, only high IPI was found significantly associated with HR of 5.6 (2.68–11.7), *p* < 0.001 (**Figure 1B**). On Kaplan-Meier survival analysis based on cell of origin, there was no effect on overall survival and EFS (**Figures 1C, D**).

**Table 2:** Univariate analysis of prognostic factors for event-free survival and overall survival.

Prognostic factor	Event-free survival		Overall survival	
	HR (95% CI)	*p*-value	HR (95% CI)	*p*-value
Age				
≤60 years				
>60 years	1.77 (1.24–2.54)	0.001	2.73 (1.62–4.58)	<0.001
Gender				
Male				
Female	1.04 (0.72–1.49)	0.82	1.28 (0.76–2.17)	0.34
B symptoms				
Absent				
Present	1.87 (1.32–2.67)	<0.001	2.19 (1.30–3.70)	0.002
ECOG PS				
0–2				
3–4	1.70 (1.19–2.42)	0.002	3.17 (1.85–5.43)	<0.001
Albumin				
≥3.5 g/dl				
<3.5 g/dl	2.04 (1.42–2.91)	<0.001	2.43 (1.42–4.13)	<0.001
LDH				
Normal				
High	1.69 (1.17–2.42)	0.03	2.54 (1.44–4.47)	0.12
Bulky disease				
No				
Yes	1.80 (1.26–2.56)	<0.001	1.44 (0.85–2.44)	0.01
Extranodal involvement				
No				
Yes	1.16 (0.81–1.66)	0.001	1.17 (0.69–1.97)	0.54
Cell of origin				
GCB				
Non-GCB	1.45 (0.96–2.17)	0.07	1.55 (0.83–2.87)	0.15
IPI				
Low risk				
Inter. risk	1.80 (1.20–2.71)		2.45 (1.23–4.89)	
High risk	2.23 (1.34–3.72)	0.015	5.60 (2.68–11.7)	<0.001
Complete response				
Yes				
No	2.25 (1.48–3.43)	<0.001	1.78 (1.16–2.78)	0.01

## Discussion

Real-world data on the outcome of patients with DLBCL treated with standard R-CHOP in developing countries are lacking due to resource limitations. We tried to analyze the outcome of DLBCL patients treated with R-CHOP over a period of 7 years at our center. The median age of our population was 48 years which is more than a decade earlier than in the Western studies (12). This trend of comparatively younger median age is seen in multiple previous studies on DLBCL as well as in other common malignancies in India (13–17). The exact reason for this trend is unknown but may be due to a higher proportion of young and middle-aged people in India or due to a referral bias toward younger patients for treatment at higher center. The male-to-female ratio of 2:1 is higher than Western studies but consistent with recent studies in India (13–17). In the present study, 30% of patients presented with poor ECOG PS (≥2) which was similar to that seen in Western studies (18). The cause of this higher proportion of patients presenting with B symptoms and extranodal disease in our study are due to late presentation resulting from a delay in diagnosis, but the exact reason is not known. The percentage of patients presenting with elevated LDH and those presenting with advanced disease was similar to Western data (18, 19). The complete response rate (CR) seen in our study was 74.9%, which is similar to that reported in landmark trials that resulted in the approval of rituximab-based chemoimmunotherapy in DLBCL (**Table 3**) (19–21).

**Table 3:** Comparative analysis of patient characteristics and response.

Study [reference]	Nimmagadda et al. (15)	Coiffier et al. (20)	Pfreundsch et al. (21)	Cunningham et al. (22)	Present study
Design	Retrospective	RCT	RCT	RCT	Retrospective
*n*	791	399	824	1,080	417
Age group	16–92	60–80	18–60	≥18	18–76
Median age	53	69	47	61	48
M:F	2:1	1:1	1.3:1	1:1	2:1
ECOG PS 0, 1	72%	78%	99%	WHO 0, 1–87%	PS 0–2 = 65%
B symptoms	38%	NA	26%	44%	43%
Bulky	NA	30%	40%	50%	35%
Raised LDH	NA	65%	30%	65%	47%
Stage 3/4	52%	79%	20%	64%	51%
Treatment	CHOP (41%) *vs*. RCHOP (43%)	R-CHOP *vs*. CHOP	R-CHOP *vs*. CHOP	RCHOP 14 *vs*. RCHOP 21	R-CHOP21
Response	66%	82% *vs*. 69%	–	91% *vs*. 88%	85%
CR	55%	75% *vs*. 63%	86% *vs*. 68%	58% *vs*. 63%	74.9%
PD	10%	10% *vs*. 22%	4% *vs*. 11%	4% *vs*. 6%	7.2%

RCT, randomized control trial; n, number of patients; M:F, male:female ratio; PS, performance status; LDH, lactate dehydrogenase; CR, complete response; PD, progressive disease; EFS, event-free survival; OS, overall survival; R-CHOP, rituximab, cyclophosphamide, doxorubicin, vincristine, and prednisolone; NA, not available.

When comparing with Indian data, our outcome appears better than most previously reported series on DLBCL; however, most of these prior studies also included patients who received non-rituximab-based chemotherapy (13–17). A recent study comparing the outcome of DLBCL patients treated with biosimilars vs. original rituximab (MabThera^®^, Roche, Basel, Switzerland) reported a 5-year OS of 81% and also found that outcome is similar regardless of whether biosimilars or original rituximab is used (23, 24). The 3-year OS of 88% seen in our study is comparable with other studies from India in the rituximab era (15, 24, 25). Although long-term follow-up is needed, we may conclude that when treated with standard R-CHOP chemotherapy, the outcome of Indian patients is almost similar to real-world Western data (15).

Previous studies have shown that the prognosis of GCB subtype DLBCL is better than the non-GCB subtype (25–28). There are no studies from our population assessing the prognostic significance of COO in the rituximab era. In the current report of 417 patients, we were able to classify 90% of patients to GCB and non-GCB subgroups based on the Hans algorithm. Subtyping of the remaining 10% was not possible due to inadequate sample or logistic reasons. Although the CR rate was numerically higher with the GCB subtype, the difference was not statistically significant. EFS and OS were also not statistically different between the two COO subgroups (3-year OS 88% vs. 83.7%; *p* = 0.15). The reason for this discrepancy from Western literature may be due to the fact that classification based on Hans algorithm was not possible in 10% of cases and relatively short follow-up. Recent studies have suggested that algorithms based on IHC has poor prognostic and predictive value; instead, tests like Lymph2Cx have a concordance rate of 96% with gene expression profiling (29, 30).

The incidence of grade ≥3 neutropenia and febrile neutropenia seen in our study was 13% and 9%, respectively, which is lower when compared with reported studies (31). This may be because our patient population is predominantly younger and the majority present with good risk International Prognostic Index score.

## Limitations

The major drawback of our study was a proportion of patients (10%) who were lost to follow-up. This may have resulted in a biased overall survival seen in our study. Grade I/II toxicities were not reliably recorded. Other inherent limitations of the study include its retrospective data collection.

## Conclusion

This is the first study from India evaluating the impact of COO on outcomes of DLBCL patients when treated with uniform RCHOP protocol. The outcome of patients with DLBCL treated with standard RCHOP chemotherapy is excellent in our patient population. The cell of origin of DLBCL has no impact on CR, EFS, and OS if the patients are appropriately treated with standard doses and frequency of RCHOP. RCHOP is well tolerated in our patients and results are comparable with the Western data.

## Data Availability Statement

The raw data supporting the conclusions of this article will be made available by the authors, without undue reservation.

## Ethics Statement

The study was conducted according to the ethical guidelines established by the Declaration of Helsinki, and this study was approved by the Institutional Ethics Committee for Post-Graduate Research for Clinical Science.

## Author Contributions

All authors listed have made a substantial, direct, and intellectual contribution to the work and approved it for publication.

## Conflict of Interest

The authors declare that the research was conducted in the absence of any commercial or financial relationships that could be construed as a potential conflict of interest.

## Publisher’s Note

All claims expressed in this article are solely those of the authors and do not necessarily represent those of their affiliated organizations, or those of the publisher, the editors and the reviewers. Any product that may be evaluated in this article, or claim that may be made by its manufacturer, is not guaranteed or endorsed by the publisher.
